# A Mouse Model of Lipoatrophy Reveals Relationships Between Beige Fat Appearance and Female Fertility

**DOI:** 10.1210/endocr/bqaf164

**Published:** 2025-11-05

**Authors:** Elizabeth S Anaya, William Dion, Pradip K Saha, Aaron R Cox, Evelyn de Groot, Avery A Ahmed, Jessica B Felix, Bokai Zhu, Stephanie A Pangas, Sean M Hartig

**Affiliations:** Division of Diabetes, Endocrinology, and Metabolism, Baylor College of Medicine, Houston, TX 77030, USA; Department of Medicine, Baylor College of Medicine, Houston, TX 77030, USA; Graduate Program in Cancer and Cellular Biology, Baylor College of Medicine, Houston, TX 77030, USA; Aging Institute of UPMC, University of Pittsburgh School of Medicine, Pittsburgh, PA 15219, USA; Division of Diabetes, Endocrinology, and Metabolism, Baylor College of Medicine, Houston, TX 77030, USA; Department of Medicine, Baylor College of Medicine, Houston, TX 77030, USA; Center for Metabolic and Degenerative Diseases, the Brown Foundation Institute of Molecular Medicine for the Prevention of Human Diseases, University of Texas Health Science Center at Houston, Houston, TX 77030, USA; Division of Diabetes, Endocrinology, and Metabolism, Baylor College of Medicine, Houston, TX 77030, USA; Department of Medicine, Baylor College of Medicine, Houston, TX 77030, USA; Graduate Program in Cancer and Cellular Biology, Baylor College of Medicine, Houston, TX 77030, USA; Graduate Program in Development, Disease Models & Therapeutics, Baylor College of Medicine, Houston, TX 77030, USA; Department of Pathology and Immunology, Baylor College of Medicine, Houston, TX 77030, USA; Division of Diabetes, Endocrinology, and Metabolism, Baylor College of Medicine, Houston, TX 77030, USA; Department of Medicine, Baylor College of Medicine, Houston, TX 77030, USA; Aging Institute of UPMC, University of Pittsburgh School of Medicine, Pittsburgh, PA 15219, USA; Division of Endocrinology and Metabolism, Department of Medicine, University of Pittsburgh School of Medicine, Pittsburgh, PA 15213, USA; Graduate Program in Cancer and Cellular Biology, Baylor College of Medicine, Houston, TX 77030, USA; Graduate Program in Development, Disease Models & Therapeutics, Baylor College of Medicine, Houston, TX 77030, USA; Department of Pathology and Immunology, Baylor College of Medicine, Houston, TX 77030, USA; Department of Molecular and Cellular Biology, Baylor College of Medicine, Houston, TX 77030, USA; Division of Diabetes, Endocrinology, and Metabolism, Baylor College of Medicine, Houston, TX 77030, USA; Department of Medicine, Baylor College of Medicine, Houston, TX 77030, USA; Graduate Program in Cancer and Cellular Biology, Baylor College of Medicine, Houston, TX 77030, USA; Graduate Program in Development, Disease Models & Therapeutics, Baylor College of Medicine, Houston, TX 77030, USA; Department of Molecular and Cellular Biology, Baylor College of Medicine, Houston, TX 77030, USA

**Keywords:** adipose tissue, reproduction, fertility

## Abstract

White adipose tissue (WAT) performs vital metabolic and endocrine functions, but roles in female reproduction remain understudied and poorly understood. Here, we report that female mice experiencing progressive lipoatrophy after knockout of *Ubc9* in adipocytes (*Ubc9^fKO^*) displayed disrupted estrous cycles, reduced ovarian reserve, and subfertility. During aging, female *Ubc9^fKO^* mice lose subcutaneous WAT more quickly than their male counterparts and weigh less than littermate controls. Subcutaneous WAT excised from female *Ubc9^fKO^* mice strongly enriched for thermogenesis genes generally associated with metabolic benefits. Female *Ubc9^fKO^* mice exhibited hypermetabolism and accumulated thermogenic, Uncoupling Protein 1-expressing beige fat cells in residual subcutaneous WAT depots in a sex-dependent manner. However, remnant beige fat appearance occurred at the expense of fertility in *Ubc9^fKO^* female mice. A high-fat diet diminished the appearance of beige fat cells and restored estrous cycle regularity among *Ubc9^fKO^* mice compared to littermate controls, despite the presence of profound insulin resistance. Together, these results reveal sexual dimorphism in a mouse model of lipoatrophy and the importance of WAT for sustaining reproduction in female mice. These findings also provide evidence that beige adipocytes compensate for fat loss at the expense of fecundity in female mice and identify pathways to improve fertility in very lean and lipodystrophic women.

White adipose tissue (WAT) responds to the body's metabolic demands, storing excess energy as triglycerides during nutrient overload and releasing free fatty acids in times of nutrient deficit (1). In females, altered body composition increases the risk of infertility. Excessive adipose tissue accumulation in overweight or obese individuals frequently associates with ovulatory disorders and decreased success in fertility treatments (2). Reduced fertility also occurs in individuals with diminished adipose tissue, such as athletes, women with lipodystrophies, or those with eating disorders, including anorexia and bulimia nervosa (2, 3). These observations demonstrate that maintaining a healthy, functional adipose tissue mass is required to sustain metabolic homeostasis and reproductive success.

Considerable evidence indicates that the amount of thermogenic beige adipocytes within subcutaneous WAT (sWAT) depots corresponds with insulin sensitivity and protection against the comorbidities of obesity and aging (4). On the contrary, some heterogeneous disorders characterized by fat atrophy couple thermogenesis in WAT and hypermetabolism with insulin resistance (5-10). It is still unclear whether beige fat recruitment can ever be translated into effective treatments for obesity. Some studies also suggest that increased beige fat activation and a hypermetabolic state can be harmful when the body is under severe metabolic stress (11) and disease states (12, 13). However, the implications of beige fat activity on female fertility remain completely unknown. Females accumulate adipose tissue differently than males, favoring storage in subcutaneous rather than visceral depots, a distribution that confers greater metabolic flexibility and insulin sensitivity (14). Subcutaneous depots harbor smaller adipocytes and display a higher potential for beiging. The depot-specific distribution, combined with estrogen signaling, results in a greater capacity for thermogenic activity within brown and white fat depots in women (15-17). Yet, the consequences of beige fat cell recruitment on female reproduction, including cycle regularity, have not been explored.

Here, we explore sex-specific metabolic responses to fat loss and the female reproductive outcomes using a lipodystrophic mouse model (*Ubc9^flox/flox^*; *Adipoq*-Cre; *Ubc9^fKO^*) (18). Previously, we showed that the small ubiquitin-like modifier E2-conjugating enzyme *Ubc9* is important for WAT expansion and the maintenance of mature white adipocytes during postnatal growth (18). In this current study, we initially focused on the long-term metabolic outcomes resulting from a female-specific increase in sWAT beige fat resulting from WAT loss. However, we surprisingly observed that female *Ubc9^fKO^* mice also have reduced fertility. To these points, our studies reveal significant consequences of sex-specific beige fat recruitment on female reproductive functions.

## Materials and Methods

### Sex as a Biological Variable

Our experiments included male and female animals for some studies, and sexually dimorphic effects are reported. The primary focus of the study was estrous cycling and reproduction in females, justifying the emphasis on one sex.

### Animal Studies

Experimental animals received humane care according to criteria in the “Guide for the Care and Use of Laboratory Animals” (8th edition, revised 2011). All experiments adhered to ARRIVE Guidelines. Experimental animals were housed (no more than 4 per cage) in a barrier-specific pathogen-free animal facility with a 12 hours dark-light cycle and free access to water and normal chow (Standard Rodent Diet [5V5R] Lab Diet Picolab Select Rodent 50 IF/9F) unless otherwise specified. *Ubc9^fl/fl^* mice used in this study were previously generated and crossed with *Adipoq-Cre* (Jackson Laboratory #028020) to generate adipocyte-specific *Ubc9* knockout (*Ubc9^fKO^*) and littermate controls (*Ubc9^fl/fl^*), previously published as *Ube2i^a-KO^* (18). Experiments were conducted using mice maintained on a C57BL/6J background and generated from *Ubc9^fl/fl^* females mated to *Adipoq-Cre* males. *Cre* transgenic mice were genotyped according to protocols provided by the Jackson Laboratory. At the end of each experiment, mice were euthanized by cervical dislocation while under isoflurane anesthesia. All female mice were euthanized in the morning (8 Am-10 Am) of diestrus for tissue and serum analysis. After euthanasia, tissues were collected and either fixed in 10% formalin or flash-frozen in liquid N2 and stored at −80 °C until use.

### RNA Isolation and qPCR

Total RNA was extracted using the RNeasy Lipid Tissue kit (Qiagen). cDNA was synthesized from total RNA using SuperScript IV VILO Master Mix (Invitrogen # 11766050). Relative mRNA expression was measured with SsoAdvanced Universal Probes Supermix reactions (Bio-Rad #175284) read out with a QuantStudio 3 real-time PCR system (Applied Biosystems). TATA-box binding protein was the invariant control. Roche Universal Probe Gene Expression Assays (mouse) or individual TaqMan Gene Expression Assays (Thermo Fisher) were used as previously described (19).

### Indirect Calorimetry

*Ubc9^fKO^* mice and littermate controls (*Ubc9^fl/fl^*) were maintained on normal chow or high-fat diet and housed at room temperature in Comprehensive Lab Animal Monitoring System (CLAMS) home cages (Columbus Instruments). Oxygen consumption, carbon dioxide production, energy expenditure (EE), food and water intake, and activity were measured over 6 days (BCM Mouse Metabolic and Phenotyping Core). Mouse body weight was recorded, and body composition was measured by magnetic resonance imaging (Echo Medical Systems) before indirect calorimetry. The first 2 days (48 hours) of measurements were excluded from the analysis to allow for the acclimation of CLAMS cages. Statistical analysis of mass-dependent variables (oxygen consumption, carbon dioxide production, EE, and food and water intake) was performed by analysis of covariance (ANCOVA) with lean body mass as a covariate and ANOVA for mass-independent variables (wheel running, respiratory exchange ratio, and physical activity) using the CalR web-based tool and standards defined by the International Indirect Calorimetry Consensus Committee (20, 21).

### Glucose and Insulin Tolerance Tests

To assess glucose tolerance, mice were fasted for 16 hours, and glucose (1 g/kg body weight) was administered by intraperitoneal injection. To assess insulin tolerance, mice were fasted for 4 hours before insulin intraperitoneal injection (0.75 U/kg body weight). Blood glucose levels were measured by a handheld glucometer.

### ELISAs and Serum Analysis

Serum collected from ad-libitum-fed, diestrus phase-matched mice was used to measure insulin (Millipore #EZRMI-13K, RRID:AB_2783856), leptin (Crystal Chem #90030, RRID:AB_2722664), adiponectin (Thermo Fisher #KMP0041, RRID:AB_2922953), and free fatty acids (ZenBio #sfa-1). The serum was also analyzed for triglycerides (Thermo Fisher TR22421) and total cholesterol (Thermo Fisher TR13421).

### Histology

Formalin-fixed, paraffin-embedded adipose sections and liver were stained with hematoxylin and eosin (H&E) by the BCM Human Tissue Acquisition and Pathology Core. Images were captured (20×) using a Nikon Ci-L Brightfield microscope.

### Immunohistochemistry

Immunohistochemistry was performed on formalin-fixed, paraffin-embedded adipose tissue sections by the BCM Human Tissue Acquisition and Pathology Core for the beige adipocyte marker UCP1 (abcam ab10983, RRID:AB_2241462).

### RNA Sequencing

Tissue was weighed, and 30 to 50 mg of tissue was used for RNA extraction. RNA was extracted using a RNeasy Lipid Tissue kit (Qiagen: 74804) along with a RNase-free DNase set (Qiagen: 79254) for DNA digestion. Library generation and sequencing were performed in the Health Sciences Sequencing Core at the University of Pittsburgh Medical Center, Children's Hospital of Pittsburgh, Rangos Research Center. RNA was assessed for quality using an Agilent TapeStation 4150/Fragment Analyzer 5300, and RNA concentration was quantified on a Qubit FLEX fluorometer. Libraries were generated with the Illumina Stranded Total Library Prep kit (Illumina: 20040529) according to the manufacturer's instructions. Library quantification and assessment were done using a Qubit FLEX fluorometer and an Agilent TapeStation 4150/Fragment Analyzer 5300. Sequencing was performed on an Illumina NextSeq 2000, using a P3 flow cell with read lengths of 2 × 101 bp, with a target of 80 million reads per sample. Sequencing data were demultiplexed by the on-board Illumina DRAGEN FASTQ Generation software (v3.10.12). Raw reads were processed and aligned to the mm10 reference genome, reads were counted with *htseq-count* (22), FPKM values were determined with *Cufflinks* (23), and differential gene expression analysis was performed with *DESeq2* (24). Data are available through the NCBI Gene Expression Omnibus (GSE289065).

### Fertility Analysis

To test fecundity, sexually mature (8-week-old) *Ubc9^fKO^* females or control littermates (*Ubc9^fl/fl^*) were pair-housed with sexually mature (8-week-old) C57BL/6J wild-type males and continuously mated for 6 months. An n = 10/group started the fertility study, but only mice n = 8,9/group survived to completion and were analyzed due to pregnancy complications resulting in animal welfare concerns. Females were monitored daily for new litters, and the number of pups, date of birth, and sex of each pup were recorded. Any resulting litters were weaned and weighed at 3 weeks of age. For estrous cycle monitoring, mice were individually housed, and vaginal lavage and cytology were performed daily for 1 month as described (25).

### Superovulation Experiments

Female mice aged 5 weeks were injected with 5 IU pregnant mare serum gonadotropin (ProSpecBio), and 46 hours later, injected with 5 IU human chorionic gonadotropin (Pregnyl; Merck Pharmaceuticals) by intraperitoneal injection. Mice were euthanized 16 to 18 hours after injection with human chorionic gonadotropin, and cumulus-oocyte complexes were harvested from the oviduct ampulla and incubated with 0.3 mg/mL hyaluronidase (Sigma-Aldrich) to detach cumulus cells, and MII oocytes counted.

### Reproductive Hormone Analysis

Blood was collected via cardiac puncture from isoflurane-anesthetized mice, and the serum was separated by centrifugation in microtainer collection tubes (Becton, Dickinson, and Company) and frozen at −20 °C until assayed. Mouse LH, FSH, anti-Mullerian hormone (AMH), and progesterone serum levels were analyzed in diestrus phase-matched mice via ELISA at the University of Virginia Ligand Core Facility. The assay method information is available online (https://med.virginia.edu/research-in-reproduction/ligand-assay-analysis-core/assay-methods/). AMH levels comparing normal chow vs high-fat diet in 6-month-old mice were measured using the same kit used by the University of Virginia Ligand Core Facility (AnshLabs #AL-113, RRID:AB_2783665).

### High-fat Diet Experiments

At 6 weeks of age, *Ubc9^fKO^* and *Ubc9^fl/fl^* littermate controls were placed on and continuously fed a high-fat diet (HFD) containing 60% of calories from fat, 25% from carbohydrate, and 15% from protein (Bio-Serv #S3282) for the entirety of the HFD experiments. Body weight measurements were taken weekly for 11 weeks. On the 12th week of HFD feeding, body weight and body composition were measured via magnetic resonance imaging (Echo Medical Systems), and mice were placed in CLAMS cages (see Indirect calorimetry). After indirect calorimetry, glucose and insulin tolerance were measured (see glucose and insulin tolerance tests). Estrous cycles were monitored daily for 30 days starting when the mice were 5 months old. At 6 months of age, the mice were euthanized in diestrus, and tissue and serum were collected for analysis. In total, the mice were on HFD for 18 weeks and further compared to 6-month-old age-matched mice fed normal chow (NC) containing 15% of calories from fat, 64% from carbohydrate, and 21% from protein (Standard Rodent Diet [5V5R] Lab Diet Picolab Select Rodent 50 IF/9F).

### Statistical Analysis

Unless otherwise noted, statistical analyses were performed using GraphPad Prism 9 (GraphPad Software, La Jolla, CA). A 2-tailed unpaired Student *t*-test was used for single comparisons. One-way or 2-way analysis of variance followed by post hoc tests were used for multiple comparisons. For gene expression data, statistical significance was assessed by multiple unpaired *t*-tests with a q value < 0.05. Statistical analysis of energy balance was performed by ANCOVA with lean body mass as a covariate and cumulative food intake by standard ANOVA using the CalR web-based tool (20). A power analysis was performed for all experimental methods, and sample sizes are indicated in the text and figure legends. All data are presented as mean ± SEM. Statistical significance shown as *P < .05, **P < .01, ***P < .001, ****P < .0001.

### Study Approval

All animal procedures were approved by the Institutional Animal Care and Use Committee of Baylor College of Medicine (Animal Protocol AN-6411).

## Results

### *Ubc9^fKO^* Mice Lose Adipose Tissue in a Sex- and Age-specific Manner

**Figure 1.**
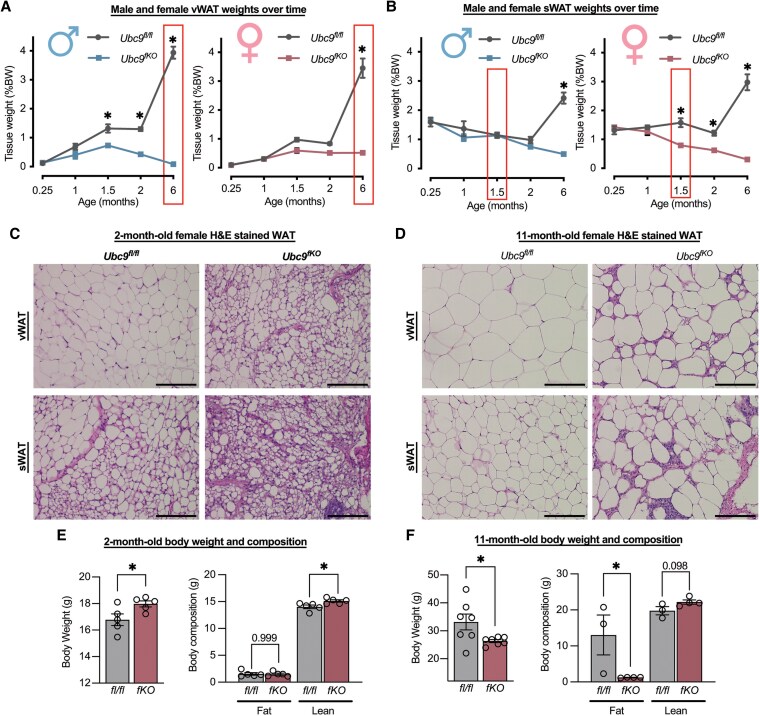
*Ubc9^fKO^* mice lose adipose tissue in a sex- and age-specific manner. (A) Visceral and (B) subcutaneous white adipose (vWAT, sWAT) weights from necropsy over time from male and female *Ubc9^fKO^* mice and *Ubc9^fl/fl^* littermate controls (n = 3-16/group). Boxes represent sex differences in WAT loss. (C) H&E staining of vWAT and sWAT white adipose tissue from 2-month-old and (D) 11-month-old female *Ubc9^fKO^* mice and *Ubc9^fl/fl^* littermate controls (n = 3-4/group). All images were taken at 20×. Scale bar set at 100 µm. (E) 2-month-old (n = 5/group) and (F) 11-month-old (n = 3-4/group) body weight and body composition measured by Echo MRI, shown as percentage of body weight. Data are mean ± SEM. *P < .05 by 2-way ANOVA followed by Fisher least significant difference test and unpaired 2-tailed Student *t*-test for body weight.

During our previous investigations of *Ubc9^fKO^* mice, we noticed sexually dimorphic metabolic phenotypes, including less sWAT inflammation and lipid accumulation in the liver of the females compared to the males. By 6 months of age, male *Ubc9^fKO^* mice are severely hyperinsulinemic, whereas *Ubc9^fKO^* females have only a marginal increase in serum insulin (18). WAT loss in both sexes reduced serum adiponectin and leptin levels, yet to a lesser degree in females (18). To investigate these observations further, we measured sWAT and visceral WAT (vWAT) tissue weights across 5 time points in male and female mice. Male and female littermate controls showed no issues with expanded body mass during standard monitoring. However, we observed notable fat loss after puberty in both sexes at the time of necropsy in *Ubc9^fKO^* mice. Fat mass varied between depot, sex, and across different time points. During the progressive fat loss, female *Ubc9^fKO^* mice did not lose all their vWAT by 6 months of age (Fig. 1A). vWAT was generally absent in male *Ubc9^fKO^* mice. However, *Ubc9^fKO^* females lost their sWAT earlier than their male counterparts, with the males having similar sWAT weights compared to littermate controls up until 6 months (Fig. 1B). These findings built on previous studies (18) and refined sex-specific responses to the metabolic stress of progressive fat loss.

Because of the sex differences in adipose tissue loss with age, we next characterized the changes in adipose tissue morphology in the vWAT and sWAT from female *Ubc9^fKO^* mice over time. We assessed the morphology starting at 2 months old, around the time when the female fat loss begins (Fig. 1A, 1B) and in 11-month-old female mice. Interestingly, at 2 months of age, *Ubc9^fKO^* females display smaller and more multilocular-appearing adipocytes in WAT depots compared to littermate controls, which were most apparent in the sWAT depot (Fig. 1C). Age also strongly affected adipose tissue morphology among genotypes. Smaller and interspersed adipocytes were abundant in 2-month-old *Ubc9^fKO^* mice relative to control littermates. However, stromal invasion and fewer hypertrophic adipocytes were observed at 11 months of age (Fig. 1D). Molecular resonance imaging (MRI) assessments of total fat and lean mass in young and aged mice showed that 2-month-old *Ubc9^fKO^* female mice weighed more than their control counterparts, likely because of increased lean mass (Fig. 1E). In comparison, older *Ubc9^fKO^* mice exhibited far less total fat mass compared to their littermates, contributing to a 20% difference in body weight (Fig. 1F).

**Figure 2.**
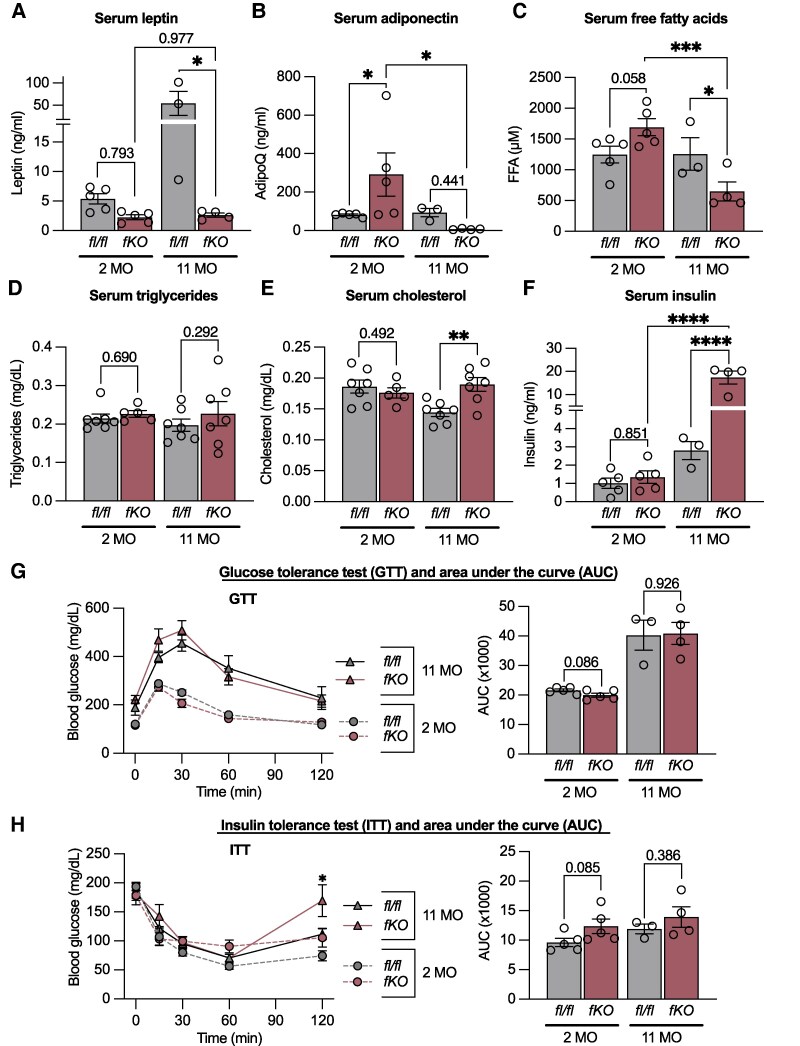
Progressive adipose tissue loss in female *Ubc9^fKO^* mice causes insulin resistance. (A) Serum leptin and (B) adiponectin (AdipoQ) from ad libitum-fed mice (n = 3-7/group). (C) Serum free fatty acids (FFA) and (D) triglycerides levels measured ad libitum (n = 3-7/group). (E) Serum cholesterol and (F) insulin (n = 3-7/group). Data are mean ± SEM. *P < .05, **P < .01, ***P < .001, ****P < .0001 by 2-way ANOVA followed by Fisher least significant difference post hoc test for serum measurements. (G) Glucose tolerance test (GTT, 1 g/kg body weight) and (H) insulin tolerance test (ITT, 0.75 U/kg body weight) with corresponding area-under-curve (AUC) measurements at 2 months (n = 5) and 11 months (n = 3-4). Data are mean ± SEM. *P < .05 by repeated measures ANOVA for GTT and ITT and unpaired 2-tailed Student *t*-test for AUC.

As a consequence of significant fat loss, we also found depleted serum levels of the adipokines leptin (Fig. 2A) and adiponectin (Fig. 2B) in 11-month-old *Ubc9^fKO^* mice but not in younger mice of either genotype. Smaller adipocytes in young *Ubc9^fKO^* mice likely contributed to higher circulating adiponectin (Fig. 2B) and free fatty acids (Fig. 2C), whereas lipoatrophy later in life demonstrably reversed or completely eliminated these effect sizes. No difference in serum triglycerides at either age was observed (Fig. 2D). Serum cholesterol and insulin levels increased by almost 50% exclusively in older *Ubc9^fKO^* mice (Fig. 2E, 2F). Surprisingly, female *Ubc9^fKO^* mice remained glucose tolerant when given 1 g/kg body weight glucose but were only mildly insulin resistant with 0.75 U/kg body weight insulin (Fig. 2G, 2H), even though hyperinsulinemia was present in older, lipoatrophic females.

### Young *Ubc9*^fKO^ Female Mice Recruit Beige Fat in Atrophied WAT

**Figure 3.**
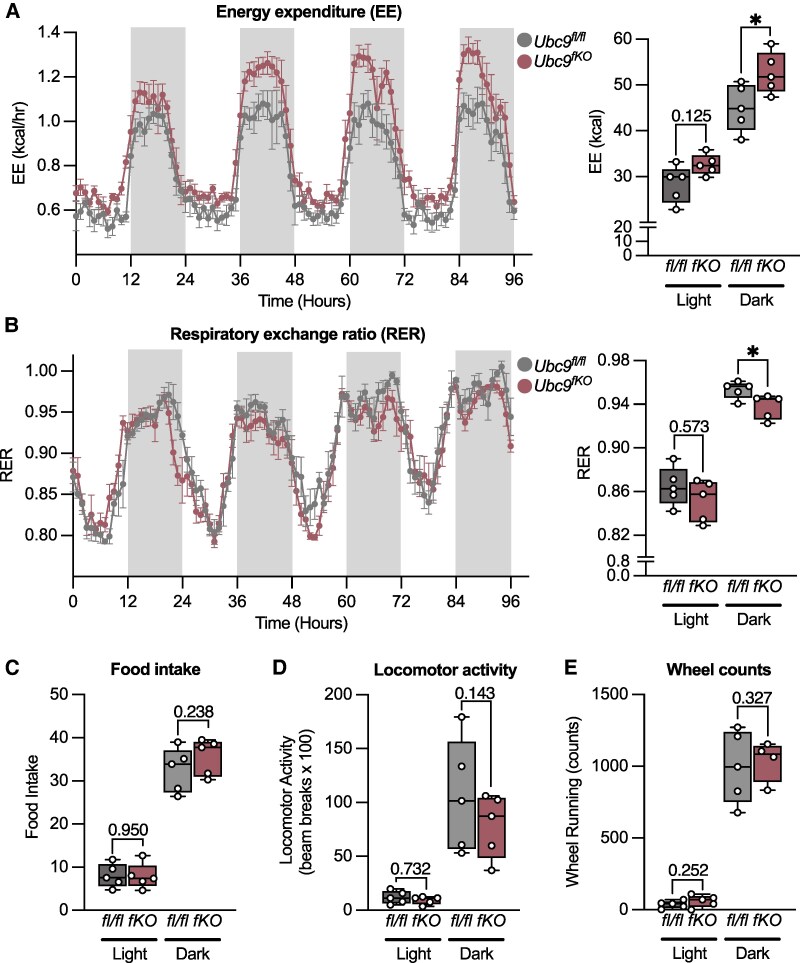
2-month-old *Ubc9^fKO^* females display hypermetabolic phenotypes. 2-month-old *Ubc9^fKO^* and *Ubc9^fl/fl^* littermate controls were individually housed and monitored in CLAMS cages during a 96-hour period. (A) Energy expenditure (EE). (B) Respiratory exchange ratio (RER). (C) Average total food intake in the light and dark phase. (D) Locomotor activity shown as beam breaks × 100. (E) Wheel running counts. Statistical analysis performed by ANCOVA with lean body mass as a covariate for body mass-dependent variables (EE and food intake) and ANOVA for mass-independent variables (RER, activity) using CalR web-based tool (n = 5/group). Data are mean ± SEM. *P < .05.

To characterize whole-body energy balance and substrate utilization during the early stages of fat loss, we placed 2-month-old female *Ubc9^fKO^* mice in CLAMS cages and explored phenotypes after correcting for lean body mass using CalR (20). Indirect calorimetry revealed hypermetabolism from increased energy expenditure (Fig. 3A). We also observed a shift in substrate utilization toward fatty acid oxidation in female *Ubc9^fKO^* mice compared to *Ubc9^fl/fl^* littermate controls, as indicated by a lower respiratory exchange ratio in the active (dark) phase (Fig. 3B). Energy balance contributions from food intake (Fig. 3C), locomotor activity (Fig. 3D), or wheel running (Fig. 3E) were similar between genotypes.

**Figure 4.**
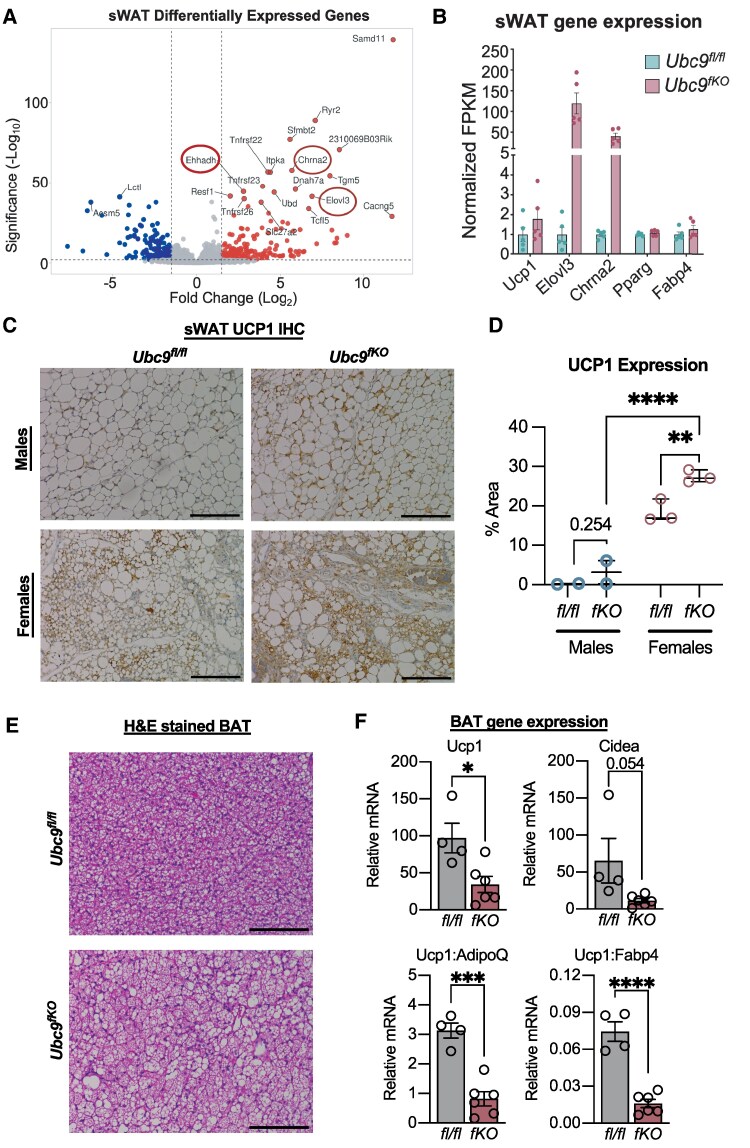
Young *Ubc9^fKO^* female mice recruit beige fat to compensate for WAT atrophy. (A) RNA-seq analysis of WAT from 2-month-old mice showing sWAT differentially expressed genes, highlighting important highly expressed genes (circles). (B) RNA expression shown as normalized FPKM in sWAT. (C) Immunohistochemistry staining of UCP1 in 2-month-old male and female *Ubc9^fKO^* mice and *Ubc9^fl/fl^* littermate controls mice and measured staining (D). All images were taken at 20×. Scale bar set at 100 µm (n = 2-3/group). (E) H&E-stained brown adipose tissue (BAT) from 2-month-old *Ubc9^fKO^* and *Ubc9^fl/fl^* littermate controls. Images taken at 20×. Scale bar set at 100 µm (n = 3/group). (F) Relative mRNA of thermogenic genes from BAT (n = 4-6/group). Data are mean ± SEM. *P < .05, **P < .01, ***P < .001, ****P < .0001, by multiple Student *t*-test for gene expression and 2-way ANOVA followed by Fisher least significant difference post hoc test for UCP1 expression.

Next, we performed bulk RNA-sequencing (RNA-seq) of whole WAT depots to determine broad gene expression changes occurring with whole-body metabolic changes among 2-month-old female *Ubc9^fKO^* mice compared to *Ubc9^fl/fl^* littermate controls. These efforts uncovered clear signatures that described the impacts of *Ubc9* knockout in the vWAT and sWAT. Kyoto Encyclopedia of Genes and Genomes analysis identified that *Ubc9^fKO^* vWAT and sWAT shared higher expression of genes enriched in pathways known to favor adipocyte functions, including PPAR signaling and lipid metabolism. Given that a lowered respiratory exchange ratio is associated with enhanced thermogenic activity and accumulation of beige fat (12, 26), we were not surprised to find marker genes of thermogenic programs (*Ehhadh, Elovl3*, *Chrna2*) among the most upregulated genes in the sWAT (Fig. 4A). Ehhadh and Elovl3 participate in fatty acid oxidation, which increases substantially during times of fasting/cold exposure and increased thermogenesis (27, 28). Additionally, Chrna2 encodes a cholinergic receptor that is important in both canonical and noncanonical beige fat activation (29). RNA-seq analysis confirmed higher expression of *Chrna2* and *Elovl3,* whereas white fat genes *Pparg* and *Fabp4* showed no effect (Fig. 4B). *Ucp1,* an important marker of thermogenesis and WAT browning, was also marginally increased in sWAT from *Ubc9^fKO^* mice (Fig. 4B).

Beige fat appearance is sexually dimorphic, and the influence of estrogen on beige/brown fat induction has been previously suggested (15, 17, 30). Females are therefore proposed to have a greater capacity for beige/brown fat activation, especially in response to dietary and environmental stressors. Likewise, sWAT beige adipocyte emergence in female *Ubc9^fKO^* may confer important adaptive responses to compensate for the loss of *Adipoq* + fat reserves. To build confidence that *Ubc9^fKO^* female mice accumulated more beige fat, sWAT from 2-month-old female mice was analyzed by immunohistochemistry for UCP1 expression and compared to littermate controls and males. We observed morphological and UCP1 expression changes in sWAT across sexes and genotypes. sWAT excised from female *Ubc9^fKO^* mice contained a greater abundance of smaller, multilocular adipocytes compared to males and littermate controls, indicative of more beige fat cells. Immunohistochemistry further confirmed that these cells were UCP1 + adipocytes in *Ubc9^fKO^* female mice (Fig. 4C). A substantial increase in UCP1 levels was observable in female knockouts compared to all other groups (Fig. 4D).

The overall contribution of beige fat to metabolic activity is considerably lower than that of brown adipose tissue (BAT) (31). As such, we determined whether the enhanced energy expenditure could be explained instead by any markers of increased BAT thermogenesis. BAT excised from 2-month-old *Ubc9^fKO^* females contained more unilocular adipocytes, apparent in H&E-stained histological sections (Fig. 4E). Additionally, reduced expression of thermogenesis genes (*Ucp1*, *Cidea*) in *Ubc9^fKO^* females (Fig. 4F) failed to explain the hypermetabolism seen in young female *Ubc9^fKO^* mice. Our findings suggest beige fat recruitment in females, but not BAT thermogenesis, contributes to hypermetabolism in this mouse model of lipoatrophy.

### Estrous Cycle Irregularity and Subfertility Precede Lipoatrophy in Female Mice

**Figure 5.**
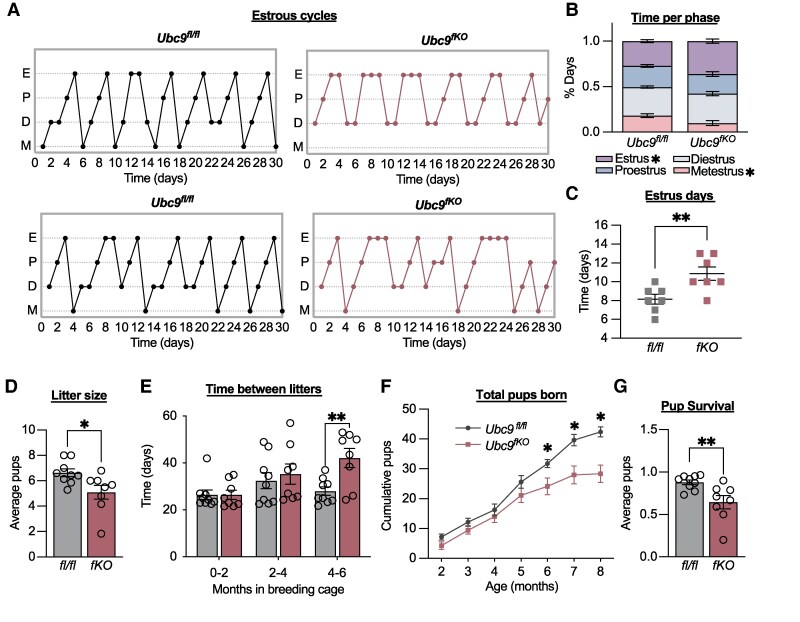
Estrous cycle irregularity and subfertility precede complete lipoatrophy in female mice. (A) Representative 30-day estrous cycle monitoring in 2-month-old *Ubc9^fKO^* and littermate controls (n = 7/group). (B) Percent days in each phase and (C) number of days spent in estrus over a 30-day period (n = 7/group). Data are mean ± SEM. *P < .05, **P < .01 by 2-way ANOVA followed by Fisher least significant difference post hoc test. (D) Overall average litter size per female over a 6-month period. (E) Time in days between litters per female separated into 2-month-long periods. Data are mean ± SEM. **P < .01, by repeated measures ANOVA. (F) Total number of pups born per female over a 6-month-long fertility study mated to WT mice starting at 2 months of age (n = 8-9/group). Data are mean ± SEM. *P < .05, by repeated measures ANOVA. (G) Pup survival measured by as a ratio between number of pups weaned and born per female. Data are mean ± SEM. *P < .05, **P < .01 by unpaired 2-tailed Student *t*-test for litter size and pup survival.

The specific impacts of recruited beige fat on female reproduction remain unknown. Given the increased energy expenditure associated with beige adipocytes within intact sWAT observed in female *Ubc9^fKO^* mice, we next investigated whether estrous cycle regularity and fertility were affected. Thirty-day estrous cycle monitoring in 2-month-old female *Ubc9^fKO^* mice revealed irregular cycles (Fig. 5A), characterized by a reduction in metestrus days, an increase in days spent in estrus (Fig. 5B), and a greater overall proportion of estrus days per month (Fig. 5C). Because *Ubc9^fKO^* displayed abnormal estrous cycles, we conducted a 6-month-long fertility assessment in which sexually mature 8-week-old *Ubc9^fKO^* and *Ubc9^fl/fl^* controls were continuously mated with age-matched wild-type (WT) males. As a result, *Ubc9^fKO^* dams had overall smaller litters, decreased by 2 pups on average (Fig. 5D). Additionally, the time between litters significantly increased over time in *Ubc9^fKO^* mice, averaging over 40 days in the last 2 months of the study (Fig. 5E). As a result, we observed a significant reduction in the total number of pups born throughout the study from *Ubc9^fKO^* dams (Fig. 5F). Interestingly, after birth, a significant number of pups did not survive past weaning age (Fig. 5G). Premature death in which pups do not survive to weaning is seen in other lipodystrophic models (32) and is often attributed to pup fat loss. However, *Ubc9^fKO^* does not generate a germline mutation, and *Ubc9^fKO^* females were bred to reproductively young WT males. Offspring are heterozygous for the *Ubc9* floxed allele only in adipocytes and nowhere else in the body. Therefore, pup attrition was likely a result of WAT dysfunction from *Ubc9^fKO^* dams, and the energy requirements associated with supporting larger litters.

**Figure 6.**
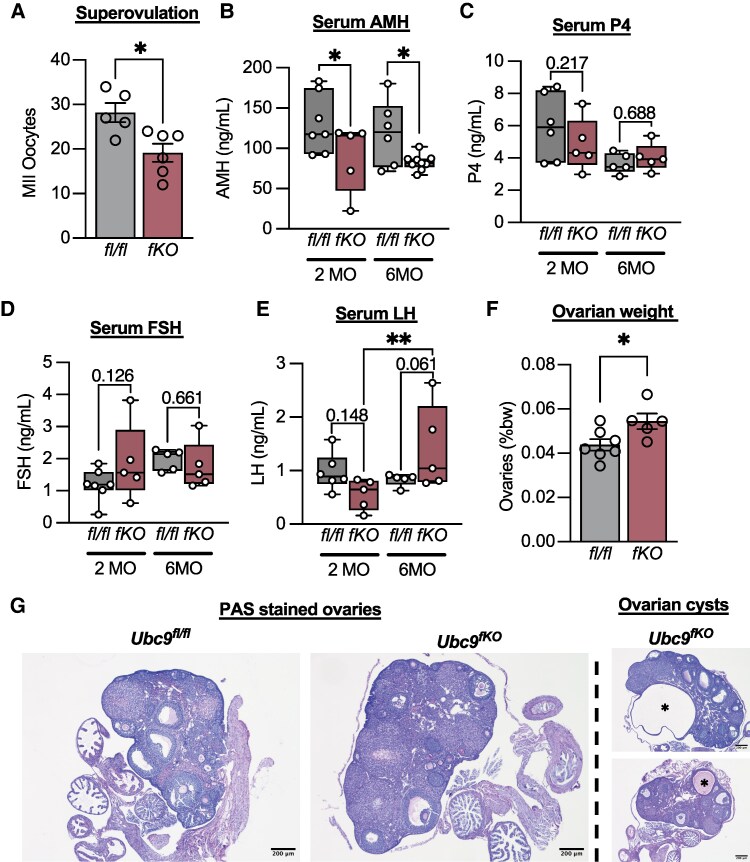
Adipose tissue loss correlates with dysfunctional ovaries. (A) Number of MII oocytes ovulated from superovulation experiment using 5-week-old *Ubc9^fKO^* and *Ubc9^fl/fl^* littermate controls (n = 5,6/group). Reproductive hormone levels (B) anti-Mullerian hormone, (C) progesterone, (D) FSH, and (E) LH measured from serum in 2-month and 6-month-old mice (n = 5-7/group). (F) Ovarian weight shown as % body weight in 6-month-old mice. (G) Periodic acid–Schiff-stained ovaries, ovarian cysts denoted with asterisks. All images taken at 4×. Scale bars represent 200 μm. Data are mean ± SEM. *P < .05, **P < .01 2-way ANOVA followed by Fisher least significant difference post hoc test and unpaired 2-tailed Student *t*-test for superovulation and ovarian weight.

To confirm that irregular estrous cycles imparted *Ubc9^fKO^* subfertility, we also conducted superovulation studies. A decrease in the number of MII oocytes retrieved from young *Ubc9^fKO^* females (Fig. 6A) indicated compromised ovulatory capacity. Likewise, both 2- and 6-month-old *Ubc9^fKO^* mice exhibited lowered levels of serum AMH (Fig. 6B), a well-established marker of the ovarian reserve that declines during aging (33), indicating lipoatrophic mice accumulated defects related to ovarian follicle dynamics. Additionally, no difference in serum progesterone and FSH were seen between genotypes (Fig. 6C, 6D). LH levels were higher in aged *Ubc9^fKO^* females but not in their littermate controls, supporting an ovarian dysfunction phenotype (34) (Fig. 6E). In addition to irregular estrous cycles, decreased ovulatory capabilities, and decreased AMH, we also observed an increase in ovarian weight in 6-month-old *Ubc9^fKO^* females (Fig. 6F). Histological analysis of periodic acid–Schiff-stained ovaries revealed an increase in the prevalence of large ovarian cysts, in which 2 of the 5 *Ubc9^fKO^* ovaries contained cysts, while none were found in the controls (Fig. 6G). Large ovarian cysts are often found with advanced ovarian age (35) and were likely driving the increased ovarian weight. Together, energy balance and fertility assessments point to compromised fecundity associated with hypermetabolism and beige fat recruitment in *Ubc9^fKO^* females.

### High-fat Diet Feeding Normalizes *Ubc9^fKO^* Estrous Cycles

**Figure 7.**
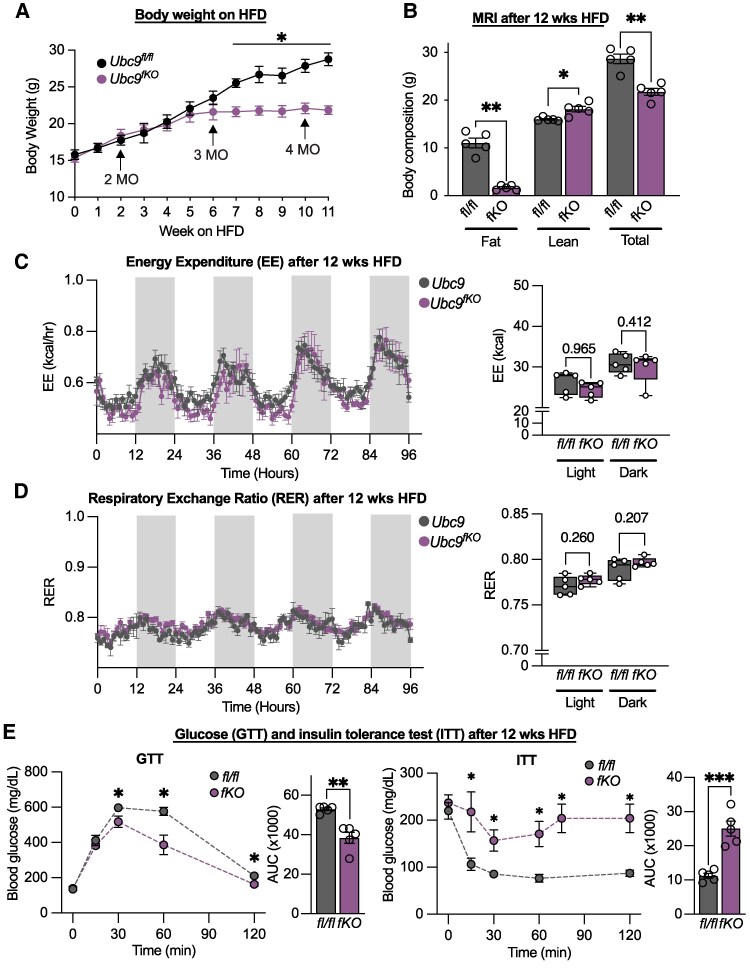
High-fat diet feeding normalizes energy expenditure in lipoatrophic mice to levels in littermate controls but worsens insulin sensitivity. (A) Body weight measurements (G) *Ubc9^fKO^* and *Ubc9^fl/fl^* on long-term HFD (n = 5,7/group). (B) Body composition measured by Echo MRI, shown as a percentage of body weight (n = 5/group). (C) Energy expenditure (EE) and (D) respiratory exchange ratio (RER) measured in CLAMS cages during a 96-hour period from *Ubc9^fKO^ and Ubc9^fl/fl^* on HFD. Statistical analysis was performed by ANCOVA with lean body mass as a covariate for body mass-dependent variables (EE) and ANOVA for mass-independent variables (RER) (n = 5/group). (E) Glucose (GTT) and insulin (ITT) tolerance test with area-under-curve (AUC) measurements from HFD-fed mice (n = 5/group). Data are mean ± SEM. *P < .05, **P < .01,***P < .001, by 2-way ANOVA followed by Fisher least significant difference post hoc, repeated measures ANOVA for GTT and ITT, and unpaired 2-tailed Student *t*-test for AUC.

**Figure 8.**
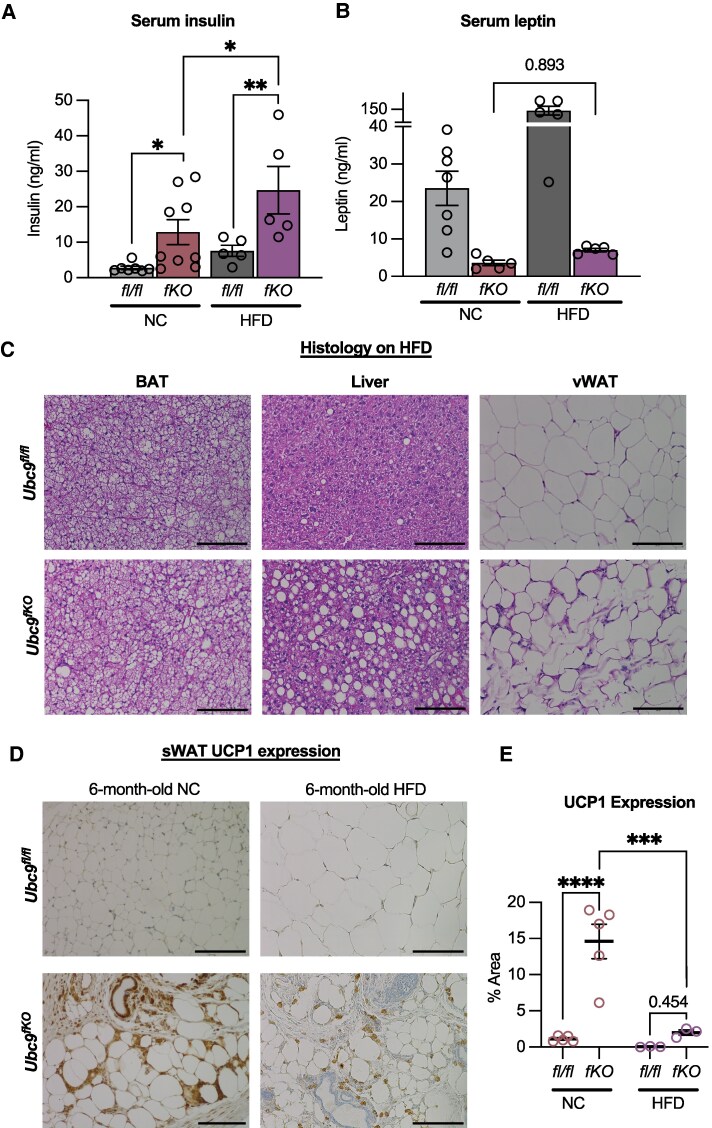
Beige fat levels in *Ubc9^fKO^* females decrease after long-term HFD feeding. (A) Serum insulin and (B) leptin levels measured postmortem in 6-month-old mice fed normal chow and after 18 weeks of HFD (n = 5-7/group). (C) Representative images of brown adipose (BAT), liver, visceral white adipose tissue (vWAT) H&E-stained histology sections from *Ubc9^fKO^* and *Ubc9^fl/fl^* mice after HFD (n = 3-4/group). Images taken at 20× with scale bars at 100 µm. (D) Immunohistochemistry staining of UCP1 (E) and immunohistochemistry quantification in subcutaneous white adipose tissue (sWAT) taken from 6-month-old female *Ubc9^fKO^* and *Ubc9^fl/fl^* littermate control mice fed normal chow (NC) or HFD. All images were taken at 20× (n = 3/group). Data are mean ± SEM. *P < .05, **P < .01, ***P < .001, ****P < .0001, by 2-way ANOVA followed by Fisher least significant difference post hoc.

A sustained HFD decreases sympathetic tone, thermogenic genes, and beige fat abundance (29). We tested the hypothesis that HFD reduces beige fat activity in *Ubc9^fKO^* and normalizes fertility phenotypes to the effect sizes observed in control littermates. *Ubc9^fKO^* females maintained similar weight gain as controls at the beginning of HFD but weighed significantly less than controls after 6 weeks of HFD (Fig. 7A). By 12 weeks of HFD, *Ubc9^fKO^* mice had significantly less fat mass and more lean mass than littermate controls (Fig. 7B). Further, CLAMS studies of *Ubc9^fKO^* females demonstrated that 12 weeks of HFD feeding effectively normalized the previously observed hypermetabolic phenotype to levels observed in *Ubc9^fl/fl^* controls. By normalizing energy expenditure (Fig. 7C) and the respiratory exchange ratio (Fig. 7D), female *Ubc9^fKO^* mice on HFD were comparable to controls despite losing fat mass. Consequently, HFD caused demonstrably worsened insulin sensitivity (Fig. 7E, insulin tolerance test), confirmed by insulin tolerance test. Surprisingly, glucose excursion (Fig. 7E, glucose tolerance test) was better in female knockouts compared to control mice on HFD, likely because of high baseline insulin levels. Serum insulin (Fig. 8A) and leptin (Fig. 8B) were raised in response to HFD in *Ubc9^fl/fl^* littermate controls. However, only serum insulin was increased in *Ubc9^fKO^* mice fed HFD, whereas leptin levels were not different compared to knockouts on NC (Fig. 8B).

The inability of WAT depots to sequester lipids, as seen in lipodystrophy and obesity phenotypes, causes ectopic accumulation of energy in peripheral organs, often in a sexually dimorphic manner (36, 37). In *Ubc9^fKO^* female mice, HFD caused profound accumulation of large fat droplets in the liver and brown adipose tissue depots (Fig. 8C). These data demonstrated that the additional HFD shifts amplifies severe metabolic dysfunction in *Ubc9^fKO^* mice. To further determine if HFD-associated metabolic dysfunction also reduced beige fat, we further analyzed histological sections of sWAT. Consistent with normalized energy expenditure profiles, sWAT UCP1 in *Ubc9^fKO^* and *Ubc9^fl/fl^* is lower after HFD compared to NC diet exposures (Fig. 8D, 8E).

**Figure 9.**
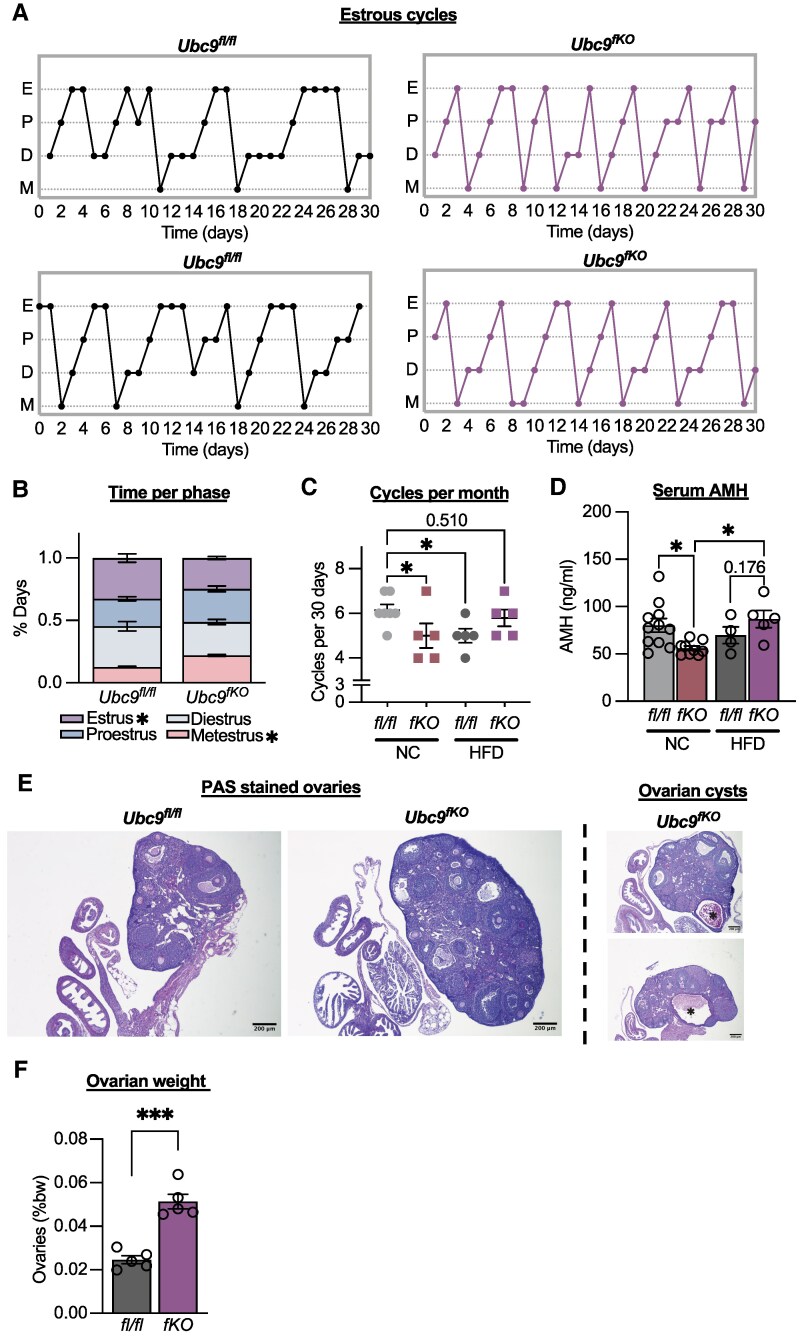
High-fat diet feeding normalizes *Ubc9^fKO^* estrous cycles. (A) Representative 30-day estrous cycle monitoring in *Ubc9^fKO^* littermate controls on HFD (n = 5/group). (B) Percent days in each phase and number of days spent in estrus over a 30-day period (n = 5/group). (C) Number of cycles in 30 days between *Ubc9^fKO^* and *Ubc9^fl/fl^* mice on NC compared to HFD. (D) Serum AMH levels measured postmortem in 6-month-old mice fed normal chow and after 18 weeks of HFD. (E) Periodic acid–Schiff-stained ovaries, ovarian cysts denoted with asterisks. All images taken at 4×. Scale bars represent 200 μm. (F) Ovarian weight shown as % body weight. Data are mean ± SEM. *P < .05, ***P < .001, by 2-way ANOVA followed by Fisher least significant difference post hoc, and unpaired 2-tailed Student *t*-test for ovarian weight.

Although *Ubc9^fKO^* females on HFD displayed worsened metabolic phenotypes, we wanted to see if lowered energy expenditure and loss of beige fat affected the estrous cycle irregularity in these mice. Remarkably, estrous cycles of *Ubc9^fKO^* were normalized, with equivalent times spent in each phase and overall cycle regularity following the HFD intervention (Fig. 9A, 9B). Countless other studies have shown that HFD feeding can result in cycle irregularity in WT mice (38). Similarly, *Ubc9^fl/fl^* controls on HFD also experienced cycle irregularity and disrupted estrous cycles. Because of the cycle irregularity displayed in control mice on HFD, we also monitored estrous cycles in age-matched mice on NC. HFD-fed *Ubc9^fKO^* mice had a comparable number of cycles per 30 days to age-matched *Ubc9^fl/fl^* mice on NC when compared by diet and genotype (Fig. 9C).

We next measured serum AMH levels in the mice after long-term HFD and compared the levels to age-matched mice fed NC. To our surprise, HFD not only improved cycle regularity, but AMH levels were higher and similar to those of controls on NC (Fig. 9D). However, HFD did not decrease the prevalence of large ovarian cysts or ovarian weight (Fig. 9E, 9F), with 2 of the 5 *Ubc9^fKO^* ovaries containing cysts. Although the presence of cysts was not decreased, our data provide strong evidence that HFD feeding and decreasing energy expenditure improved cycle regularity and AMH levels in *Ubc9^fKO^* mice.

## Discussion

In this study, we used our previously established mouse model of lipodystrophy as a tool to study how female-specific fat loss affected reproductive outcomes. We found evidence of increased WAT thermogenesis in female *Ubc9^fKO^* mice, demonstrated by higher UCP1 in sWAT and not BAT, along with other thermogenic genes and a systemic increase in energy expenditure. Prior studies demonstrated that increased thermogenesis can sustain metabolic health, despite reduced fat stores, by enhancing mitochondrial function and fatty acid utilization (39). Beige fat has been rarely studied outside the context of normal and obese women. These adaptations burn and waste energy at the cost of reproductive function, exhibited by disrupted estrous cycles, reduced ovarian reserve, and diminished fertility. These results also support the notion that females are particularly vulnerable to metabolic-reproductive tradeoffs resulting from fat loss, as shown in other studies in which female reproduction is often halted in response to metabolic stress, nutritional availability, and famine when reproduction is suppressed to preserve survival (40, 41).

To our knowledge, this is the first study to correlate beige fat responses with measures of female fecundity despite clinical studies reporting reduced fertility observed in very lean women (3). Previous studies typically and superficially characterize beige fat as beneficial, associating its presence with insulin sensitivity and protection against obesity-associated metabolic dysfunction (4, 42). However, metabolic and reproductive pathways are tightly connected, and reproductive health should be considered in conjunction with metabolic health. Moreover, females have high energetic demands to ovulate and secure pregnancy, and a diversion of wasted energy to thermogenesis may decrease the energy available for reproduction.

Leptin deficiency is a well-established hallmark of infertility resulting from lipodystrophy, and leptin add back often restores fertility in other lipodystrophic mouse models (43). Consistent with this, *Ubc9^fKO^* females accumulate less serum leptin and adiponectin, correlating with reduced adipose depots. Yet, the precise level of leptin needed for sustaining fertility is not known, and the amount produced from residual WAT in *Ubc9^fKO^* female may be permissible for cycling. Importantly, HFD intervention normalized estrous cycles and AMH levels in *Ubc9^fKO^* females without prominently raising leptin levels, providing evidence of a leptin-independent mechanism that rescues the fertility outcomes in these mice. It will now be critical to identify HFD-sensitive adipokines secreted by *Ubc9^fKO^* females that normalize estrous cycles and represent mechanisms to support fertility in very lean women.

At the molecular level, we identified increased expression of thermogenic genes including *Elovl3* and *Chrna2* that have roles in both canonical and UCP1-independent thermogenesis. Although not strictly mitochondrial genes, these genes reflect important adaptations in fatty acid elongation and cholinergic signaling pathways that are implicated in thermogenic regulation (27, 29, 44). These changes highlight broad WAT remodeling in adipose tissue fuel utilization that occurs in 2-month-old *Ubc9^fKO^ mice* before significant fat loss. Future studies are thus warranted to understand how early WAT adaptations and fuel utilization impact reproductive outcomes.

Our reproductive phenotyping indicates that the ovary is the primary site of dysfunction, in which *Ubc9^fKO^* females exhibit irregular estrous cycles, lowered AMH, reduced ovulatory capacity, and large ovarian cysts. As a result, *Ubc9^fKO^* females have reduced fecundity and smaller litter sizes. Moreover, the time between litters lengthened with age in *Ubc9^fKO^* females, likely reflecting progressive ovarian decline, consistent with diminished AMH and the presence of ovarian cysts. Although upstream contributions from the hypothalamic-pituitary-gonadal axis cannot be excluded, our findings implicate ovarian defects as the main driver of decreased fertility. As such, we were also surprised to observe a decrease in pup survival, raising the possibility of additional reproductive defects, potentially in lactation competence. Although milk spots were visible in pups from *Ubc9^fKO^* dams, suggesting that nursing was occurring, altered mammary gland morphology cannot be excluded.

Our results suggest that HFD both normalized energy expenditure and rescued estrous cycle regularity, reinforcing the idea that reproductive function is tightly linked to energy allocation in the organism. Although HFD improved estrous cyclicity, presumably by lowering EE and thermogenic activity, the resulting insulin resistance demonstrates costs to metabolic function. As such, HFD may provide short-term reproductive benefits for *Ubc9^fKO^* females but, in turn, compromise insulin action in the periphery. It is possible that prolonged metabolic stress might induce irreversible alterations in ovarian function or hypothalamic-pituitary-gonadal axis regulation, preventing complete recovery of fertility, including ovarian cyst prevalence. Future work will be needed to determine whether early nutritional or environmental interventions prevent the long-term reproductive consequences of increased energy expenditure and lean mass in females. Importantly, these results underscore the complex interplay between metabolism and reproduction and provide insight into the evolutionary prioritization of energy allocation between survival and female reproductive success.

## Data Availability

Original data generated and analyzed during this study are included in this published article or in the data repositories listed in References.

## Abbreviations

AMH: anti-Mullerian hormone
ANCOVA: analysis of covariance
BAT: brown adipose tissue
CLAMS: Comprehensive Lab Animal Monitoring System
EE: energy expenditure
H&E: hematoxylin and eosin
HFD: high-fat diet
MRI: magnetic resonance imaging
NC: normal chow
RNA-seq: RNA-sequencing
sWAT: subcutaneous white adipose tissue
vWAT: visceral white adipose tissue
WAT: white adipose tissue
WT: wild-type
